# Supplementary material for: SIFusion: Lightweight infrared and visible image fusion based on semantic injection
Source: PLoS One. 2024 Nov 6;19(11):e0307236. doi: 10.1371/journal.pone.0307236 (PMC11540218; doi:10.1371/journal.pone.0307236)
Supplement: Attachment — Submitted filename: Response to Reviewers.pdf [file pone.0307236.s002.pdf]

Response to Reviewers

PLOS ONE,

Manuscript ID: PONE-D-24-04252

SIFusion: Lightweight Infrared and Visible Image Fusion Based on Semantic Injection

Song Qian, Liwei Yang, Yan Xue and Ping Li

Corresponding Author: Ping Li

17865563003@163.com

To Senior Editor and Reviewers

Thank you for giving us the opportunity to submit a revised draft of the manuscript "SIFusion: Lightweight Infrared and Visible Image Fusion Based on Semantic Injection" for publication in the Journal of "PLOS ONE". We appreciate the time and effort that editors and the reviewers dedicated to providing feedback on our manuscript and are grateful for the insightful comments and valuable improvements to our paper. We have incorporated most of the suggestions made by the reviewers. Those changes are highlighted with red color in the manuscript. Please see below, for a point-by-point response to the reviewers' comments and concerns.

Additionally, concerning the issue of the funders' role in the research, the project leader is the first author. We kindly request the editor to help us with the necessary amendments.

Reviewer #1

The Reviewer's Comment #1

Expand on how SIFusion is novel compared to existing methods, highlighting unique features and improvements.

Response:

Thank you for your suggestion. We have made revisions to the section on semantic guidance in the related work of this paper and have marked the changes with red color.

The Reviewer's Comment #2

Provide more detailed explanations of the Semantic Injection Module (SIM) and Edge Convolution Module (ECB), including mathematical formulations.

Response:

Thank you for your suggestion. We have added the mathematical formulas for the Semantic Injection Module (SIM) and the Edge Convolutional Block (ECB), along with corresponding explanations.

The Reviewer's Comment #3

Include comparative analysis with existing image fusion methods in terms of performance metrics and computational efficiency.

Response:

Thank you for your suggestion. We have added comparative analysis with existing image fusion methods in terms of performance metrics and computational efficiency. The data can be viewed in Table 5.

It should be specifically noted that only FLFuse, TarDAL, UMF-CMGR, and the method presented in this paper are implemented based on the Pytorch architecture, while the other comparative algorithms are based on the Tensorflow 1.x architecture. The model data for these methods cannot be provided directly. Therefore, the model data is obtained through a model reconstructed based on the Pytorch architecture.

The Reviewer's Comment #4

Address the practical applicability of the method, particularly in terms of computational cost and deployment in real-world scenarios.

Response:

Thank you for your suggestion. We have made revisions to address this issue in the content of the efficiency comparison experiment.

The Reviewer's Comment #5

Enhance the experimental section with more diverse datasets to validate the effectiveness of SIFusion in various conditions.

Response:

Thank you for your suggestion. In this paper, the training set of the MSRA dataset is not directly used as the training set for the network model. Instead, data augmentation is performed by segmenting the dataset, which to some extent allows the network model to have stronger generalization capabilities. Therefore, we first conduct experimental comparisons using the LLVIP dataset. Subsequently, we also perform generalization tests on the TNO dataset and the LLVIP dataset. These datasets contain a variety of different scenarios, especially day and night scenes. Thus, we believe that the existing experiments in this paper can, to a certain extent, verify the effectiveness of SIFusion under various conditions.

The Reviewer's Comment #6

Clarify how the method improves the processing of semantic information in high-level visual tasks, with specific examples or case studies.

Response:

Thank you for your suggestion. Firstly, we illustrate the importance of semantic information for image fusion tasks and the limitations of existing methods in handling semantic information through the examples in Figure 1. Then, we introduce the design of semantic information processing in our method in the Methods section, such as the loss function for the semantic feature branch and the combination of semantic features with fusion features through SIM. Next, we demonstrate the superiority of our method by comparing it with 10 mainstream algorithms. Lastly, we validate the effectiveness of SIM through ablation experiments. In addition, we also confirm the facilitative effect of our method on advanced visual tasks through semantic segmentation experiments. Overall, we analyze the research on semantic information in our method through the above content.

The Reviewer's Comment #7

Discuss limitations and potential areas for improvement in the method to provide a balanced view.

Response:

Thank you for your suggestion. We have revised the summary section and have expanded on the content within it.

The Reviewer's Comment #8

Include a section on potential future work, exploring how the method can be extended or adapted to other applications.

Response:

Thank you for your suggestion. We have revised the summary section and have expanded on the content within it.

The Reviewer's Comment #9

Improve the clarity and structure of the paper, ensuring that technical terms and concepts are well-explained for readers unfamiliar with the topic.

Response:

Thank you for your valuable suggestion. We believe that the current manuscript has already struck a balance between professional depth and accessibility as much as possible. Specialized technical terms are all referenced with citations, allowing readers who are not familiar with the subject to gain a deeper understanding. In order to maintain the professionalism of the paper and to avoid redundancy that could arise from oversimplifying the foundational knowledge, we have decided not to make further modifications to the current

version regarding this issue. If you still have difficulties understanding any of the proprietary terms, we will make further revisions to the manuscript.

The Reviewer's Comment #10

Strengthen the conclusion by summarizing key findings and their implications for both human and machine vision applications.

Response:

Thank you for your suggestion. We have revised the summary section and have expanded on the content within it.

Reviewer #2

The Reviewer's Comment #1

Why do the authors need to stitch together infrared and visible light images before inputting them into the model instead of extracting features separately? What is the difference between these two approaches?

Response:

In the field of infrared and visible image fusion, both single-branch and dual-branch structures are among the mainstream network architectures in existing fusion methods. The dual-branch structure can extract features from two different modalities separately before fusing them, offering better interpretability. In contrast, the single-branch structure obtains shared feature information with similar information domains through the same feature extraction approach. To further achieve a lightweight network model and in conjunction with the characteristics of the semantic injection architecture, the method proposed in this paper selects a single-branch structure with fewer parameters, while the unique feature information is combined with the shared feature information through a semantic injection approach.

The Reviewer's Comment #2

The author extracted fusion features and semantic features from the source image separately, but the extraction module is the same in terms of framework. Please explain how the same module extracts two different features separately.

Response:

The two encoders used in this paper for extracting fusion features and semantic features, although they have the same network structure, do not share weight parameters. Therefore, encoders with the same structure but different parameters can learn different parameters through specific loss guidance. The encoder of the

semantic branch is guided by the target loss to learn the parameters for extracting semantic features, thereby enabling these two encoders to perform different functions.

The Reviewer's Comment #3

The ablation experiment is insufficient, and it is not enough to simply remove the module for comparison. It should be compared with other typical modules to demonstrate the effectiveness of the proposed module.

Response:

Thank you for your suggestion. We have added a comparative experiment between ECB and other typical modules in the ablation experiment section.

The Reviewer's Comment #4

The author claims in "Contributions" that the ECB module is their first introduction to the field of image fusion, which is incorrect. The ECB module was introduced in 2023 for infrared and visible light image fusion and was publicly published that year.
The reference is: CHEN Zhaoyu, FAN Hongbo, MA Meiyan, et al. FECFusion: Infrared and visible image fusion network based on fast edge convolution. Mathematical Biosciences and Engineering: MBE, 2023, 20(9): 16060-16082.

Response:

Thanks for pointing out the issues in our paper, and we have revised the wording of the manuscript.

The Reviewer's Comment #5

The bold values described in Table 7 are not bolded.

Response:

Thank you very much for pointing out the issue with Table 7 in our paper. We apologize for the oversight and promise to correct this error immediately. In the revised version of the paper, we will ensure that all mentioned important data and values are displayed in bold to clearly distinguish and highlight this key information.

The Reviewer's Comment #6

Because the author describes the network proposed as a lightweight network, but the paper does not compare network parameters and network computational complexity(FLOPs), it is recommended to add this data comparison.

Response:

Thank you for your suggestion. We have added experimental comparison data between SIFusion and ten

comparative algorithms regarding runtime, operational memory space, parameter count, weight size, and FLOPs. The data can be viewed in Table 5.

It should be specifically noted that only FLFuse, TarDAL, UMF-CMGR, and the method presented in this paper are implemented based on the Pytorch architecture, while the other comparative algorithms are based on the Tensorflow 1.x architecture. The model data for these methods cannot be provided directly. Therefore, the model data is obtained through a model reconstructed based on the Pytorch architecture.
