# Supplementary material for: SIFusion: Lightweight infrared and visible image fusion based on semantic injection
Source: PLoS One. 2024 Nov 6;19(11):e0307236. doi: 10.1371/journal.pone.0307236 (PMC11540218; doi:10.1371/journal.pone.0307236)
Supplement: Attachment — Submitted filename: Response to Reviewers.pdf [file pone.0307236.s003.pdf]

Response to Reviewers

PLOS ONE,

Manuscript ID: PONE-D-24-04252

SIFusion: Lightweight Infrared and Visible Image Fusion Based on Semantic Injection

Song Qian, Liwei Yang, Yan Xue and Ping Li

Corresponding Author: Ping Li

17865563003@163.com

To Senior Editor and Reviewers

Thank you for giving us the opportunity to submit a revised draft of the manuscript "SIFusion: Lightweight Infrared and Visible Image Fusion Based on Semantic Injection" for publication in the Journal of "PLOS ONE". We appreciate the time and effort that editors and the reviewers dedicated to providing feedback on our manuscript and are grateful for the insightful comments and valuable improvements to our paper. We have incorporated most of the suggestions made by the reviewers. Those changes are highlighted with red color in the manuscript. Please see below, for a point-by-point response to the reviewers' comments and concerns.

Additionally, concerning the issue of the funders' role in the research, the project leader is the first author. We kindly request the editor to help us with the necessary amendments.

Reviewer #3

This paper introduces SIFusion, a lightweight infrared and visible light image fusion based on semantic injection. SIFusion proposes a semantic injection module to integrate semantic information. Experimental results demonstrate its superior performance both qualitatively and quantitatively. However, I still have the following concerns about this paper:

The Reviewer's Comment #1

In the comparison experiments, the authors compare the proposed SIFusion with other ten competitors on the MSRS and LLVIP datasets, but select six competitors on the TNO dataset. To avoid subjective bias, the authors should add experiments and propose the same comparable methods on the TNO dataset.

Response:

Due to the TNO dataset being one of the earliest public datasets for infrared and visible light images in the military field, its content is mainly dominated by buildings, with only a small portion of images containing common semantic targets such as pedestrians and vehicles. Therefore, the proposed method did not perform

well on this dataset. To better demonstrate the generalization performance of the proposed method, we replaced the TNO dataset with the newer M3FD dataset, which contains multi-scene and multi-target semantic information. We compared SIFusion with twelve comparative algorithms on the MSRS, M3FD, and LLVIP datasets, and SIFusion showed good fusion performance across all of them.

The Reviewer's Comment #2

A major contribution of this work is to design a semantic injection module, thus ensuring that the fused image has rich semantic information. Fig.1 gives a schematic comparison of SeAFusion and SIFusion. Therefore, to comprehensively verify the effectiveness of the method the authors should compare some semantic-aware methods, such as SeAFusion and PSFusion, in the experiments.

Response:

We have added comparative experiments related to SeAFusion and PSFusion, and conducted an analysis of the experiments. However, the models of SeAFusion and PSFusion require a larger number of parameters and greater consumption of computational resources. In contrast, the method proposed in this paper, while maintaining a lower computational complexity, can effectively integrate information from the source images, thereby generating fused images that are more in line with the visual effects of human vision.

The Reviewer's Comment #3

In the fusion experiment with the MSRS dataset, the fusion performance of GANMcC better than CSF. However, it's performance of object detection is inferior to CSF. How can we explain this inconsistency between fusion performance and downstream task performance?

Response:

Fusion performance metrics are used to evaluate the image quality of the fusion results and do not have a direct connection with the performance of downstream tasks. It is not possible to reflect the performance of downstream tasks through the fusion evaluation metrics. Therefore, we indirectly assess the performance of the semantic expression of the fusion results through the performance of the semantic segmentation model. From the experimental results, although the metric results of the method in this paper are not as good as PSFusion, the model parameters and computational resources required for inference of the method in this paper are far less than those of PSFusion.

The Reviewer's Comment #4

There are some errors in the references. For example, Ref.14 and Ref.37 were published in 2019 and 2022, respectively. Please double-check the whole manuscript.

Response:

Thank you for the reviewer's comments; we have re-examined the manuscript and made corrections to the relevant issues.
