# Supplementary material for: SIFusion: Lightweight infrared and visible image fusion based on semantic injection
Source: PLoS One. 2024 Nov 6;19(11):e0307236. doi: 10.1371/journal.pone.0307236 (PMC11540218; doi:10.1371/journal.pone.0307236)
Supplement: Attachment — Submitted filename: Response to Reviewers.pdf [file pone.0307236.s004.pdf]

Response to Reviewers

PLOS ONE,

Manuscript ID: PONE-D-24-04252

SIFusion: Lightweight Infrared and Visible Image Fusion Based on Semantic Injection

Song Qian, Liwei Yang, Yan Xue and Ping Li

Corresponding Author: Ping Li

17865563003@163.com

To Senior Editor and Reviewers

Thank you for giving us the opportunity to submit a revised draft of the manuscript "SIFusion: Lightweight Infrared and Visible Image Fusion Based on Semantic Injection" for publication in the Journal of "PLOS ONE". We appreciate the time and effort that editors and the reviewers dedicated to providing feedback on our manuscript and are grateful for the insightful comments and valuable improvements to our paper. We have incorporated most of the suggestions made by the reviewers. Those changes are highlighted with red color in the manuscript. Please see below, for a point-by-point response to the reviewers' comments and concerns.

Additionally, concerning the issue of the funders' role in the research, the project leader is the first author. We kindly request the editor to help us with the necessary amendments.

Reviewer #3

The authors have addressed most of my concerns, but there are still some minor issues that need to be addressed.

The Reviewer's Comment #1

The response reveals that the proposed method performed inadequately on the TNO dataset. However, concerning the MSRS, M3FD, and LLVIP datasets, the proposed SIFusion still exhibits suboptimal performance in comparison with SeAFusion and PSFusion. Hence, we suggest that the authors incorporate a Discussion section to elucidate this reason, thereby providing readers with more space for reflection.

Response:

Thank you for the reviewer's suggestions. We have added a section titled "Performance Discussion on the TNO Dataset" following the "Application of Semantic Segmentation" subsection to further discuss the shortcomings and reasons of the algorithm presented in this paper when applied to the TNO dataset.

The Reviewer's Comment #2

There is a lack of introduction and analysis of some key works in this field.
[1] Multi-interactive feature learning and a full-time multi-modality benchmark for image fusion and segmentation.
[2] Infrared and visible image fusion via interactive compensatory attention adversarial learning.
[3]CDDFuse: Correlation-driven dual-branch feature decomposition for multi-modality image fusion.

Response:

We have added introductions and analyses of these three key works in the section of related work.
